# Knowledge translation approaches to implement guidelines? Plan, assess, tailor, and learn

**DOI:** 10.1186/1710-1492-6-S4-A7

**Published:** 2010-12-10

**Authors:** Francine M Ducharme

**Affiliations:** 1 Research Centre, CHU Sainte-Justine and Department of Pediatrics, University of Montréal, Montréal, Québec, H3C 3J7, Canada

## 

Passive dissemination of guidelines to health care professionals is insufficient to change practice. Three weeks after the mailing of national *Asthma Diagnosis and Treatment Guidelines* to New Zealand general practitioners, only 46% of survey responders were able to locate the received guidelines, 12% had read them in detail, and only 20% indicated that it would change their practice [1]. In the face of information overload and guideline burnout among physicians [2], there is evidence that active dissemination with a simple actionable message may be more effective [3]. The Knowledge-to-Action cycle (see Figure 1 below) promoted by Graham and colleagues and strongly endorsed by the Canadian Institutes of Health Research, provides a framework for effective active dissemination [4]. It can be conceptualised in four main steps - namely planning, assessing, tailoring and learning.

**Figure 1:**
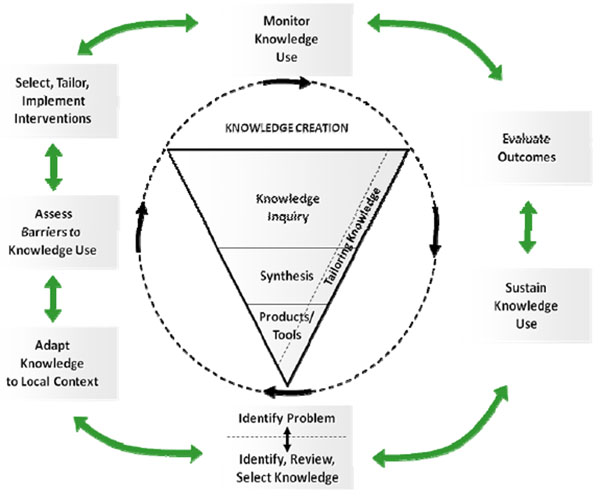
(from Ref. [4])

The planning phase involves 1) selecting one or a few key messages as priorities for implementat**i**on from the list of guideline recommendations (*e.g.,* long-term daily controller medication for children with asthma); 2) identifying the target population of health care professionals (*e.g.,* general practitioners) and settings (*e.g.,*, community practice); 3) adapting the message to the target audience (*e.g.,* prescribing by physicians; verifying adherence by pharmacists; and patient understanding of the role, safety and side effects of asthma medications by educators); and 4) selecting the action(s) to be taken and the outcomes to be measured to document adherence to the target implementation priority and its health impact.

The assessment phase includes: 1) assessing the baseline status of implementation of the selected priority(ies) preferably using objective, rather than reported, uptake by the target audience (for example, reviews of medical charts or prescriptions are superior to reported actions, which are influenced by the social desirability bias); the objective assessment of implementation may be done pre- and post-intervention or in an iterative fashion, sometimes by interrupted time series analysis, to document not only the impact of an intervention but also the sustainability of the implementation intervention. 2) Secondly, the intention to implement the specific action is an important guide to predict action. Indeed, in a large systematic review, by simply asking the target audience Sheehan discovered that 97% of those who did not intend to implement a specific action never did, while only 53% of those who intended to take the action actually did [5]. This is important as the barriers are different for intenders and non-intenders. According to the Cabana taxonomy [6,7], non-intenders face seven internal barriers related to beliefs, knowledge and attitudes and three external barriers affecting health care professionals’ ability to conform, namely barriers related to patient, guideline and environmental factors. For intenders, the intention-behaviour gap results from two main problems that can be addressed, failing to get started and getting derailed. It is critical to assess the barriers and facilitators faced by the target audience as well as the potential solutions proposed ideally by the target audience, in order to tailor the KT intervention. The omission of the assessment step is believed to explain the low success rate of a variety of KT interventions, which hovers around 10% [8].

Tailoring the KT intervention, by selecting both the KT strategy and change theory^9^ that best fit the target audience, is thus critical. The *Cochrane Effective Practice and Organization of Care Review Group* is an outstanding source of reference to select KT interventions, displaying summary estimates for various interventions tested by randomized controlled trials [8-11]. Unfortunately, it is far easier to change intention than it is to change behaviour [12]. The use of action theories to bridge the intention-behaviour gap has been well described [13]. For example, implementation intentions also called the “if-then plan” has been shown to significantly improve goal attainment [12]. It consists of four steps: identifying the self-regulatory problem Y (seeing a patient with poorly controlled asthma); identifying a cognitive/behavioural response X that would help resolve the problem (write a prescription of inhaled corticosteroids); identifying a good opportunity to instigate the response, serving as a cue (asthma quiz score of two or more filled in the clinic setting) [14]; and making a plan by generating in writing a contingency plan - if it is a situation Y, then I will do X (if I see a patient with poorly controlled asthma, that is, with an asthma quiz score of two or more, I will write a prescription for the inhaled corticosteroids) [14].

Finally, both uptake and outcome measures should be monitored for sustainability. We should learn from successes and failures as the knowledge to action cycle implies improvement through iterative rotation around the cycle. Ideally, the intervention should be tested in the context of a randomized controlled trial to best assess the impact of the intervention; because of the likelihood of contamination between health care professionals working in the same setting (clinic, hospital, *etc*.), cluster randomisation may be ideal to address this issue [15]. Whenever possible, having a third arm to examine barriers and facilitators to the uptake of the intervention is useful to better learn from our endeavour. Alternatively, such qualitative analysis of barriers and facilitators can be done after a successful or failed intervention to understand the mechanistic pathway.

In summary, the Knowledge-to-Action cycle provides the framework for designing and testing effective intervention strategies to improve implementation of guidelines by any audience, including health care professionals. The key decision remains to select a simple actionable message.

## References

[B1] Martin IR Reid JJ Dissemination of guidelines on medical practice N Z Med J 2003 116 U312 12601431

[B2] Hibble A Kanka D Pencheon D Pooles F Guidelines in general practice: the new Tower of Babel? Brit Med J 1998 317 862 3 9748185 10.1136/bmj.317.7162.862 PMC31097

[B3] Lavis JN Lomas J Hamid M Sewankambo NK Assessing country-level efforts to link research to action Bulletin of the World Health Organization 2006 16917649 10.2471/blt.06.030312 PMC2627430

[B4] Graham ID Logan J Harrison MB Lost in knowledge translation: Time for a map? J Contin Educ Health Prof 2006 26 13 24 10.1002/chp.47 16557505

[B5] Sheeran P Intention-behavior relations: A conceptual and empirical review European Review of Social Psychology 2002 12 1 36 10.1080/14792772143000003

[B6] Cabana MD Rand CS Powe NR Why don't physicians follow clinical practice guidelines? A framework for improvement JAMA 1999 282 1458 65 10.1001/jama.282.15.1458 10535437

[B7] Espeland A Baerheim A Factors affecting general practitioners' decisions about plain radiography for back pain: implications for classification of guideline barriers--a qualitative study BMC Health Services Research 2003 3 8 10.1186/1472-6963-3-8 12659640 PMC153534

[B8] Grimshaw JM Thomas RE MacLennan G Effectiveness and efficiency of guideline dissemination and implementation strategies Health Technology Assessment 2004 8 iii 72 14960256 10.3310/hta8060

[B9] Eccles M Grimshaw J Walker A Johnston M Pitts N Changing the behavior of healthcare professionals: The use of theory in promoting the uptake of research findings J Clin Epidemiol 2005 58 107 12 10.1016/j.jclinepi.2004.09.002 15680740

[B10] Eccles M Steen N Grimshaw J Effect of audit and feedback, and reminder messages on primary-care radiology referrals: A randomised trial Lancet 2001 357 1406 9 10.1016/S0140-6736(00)04564-5 11356439

[B11] Grimshaw JM Shirran L Thomas R Changing provider behavior: An overview of systematic reviews of interventions Med Care 2001 39 II2 45 10.1097/00005650-200108002-00002 11583120

[B12] Gollwitzer PM Sheeran P Implementation intentions and goal achievement: A meta-analysis of effects and processes Advances in Experimental Social Psychology 2006 38 69 119 10.1016/S0065-2601(06)38002-1

[B13] Ajzen I The theory of planned behavior Organizational Behavior and Human Decision Processes 1991 50 179 211 10.1016/0749-5978(91)90020-T

[B14] Ducharme FM Davis GM Noya F Rich H Ernst P The Asthma Quiz for Kidzä: A validated tool for children to appreciate their level of asthma control Can Resp J 2004 11 8 541 546 15611802 10.1155/2004/783740

[B15] Grimshaw J Eccles M Campbell M Elbourne D Cluster randomized trials of professional and organizational behavior change interventions in health care settings The Annals of the American Academy 2005 599 71 93 10.1177/0002716205274576

